# Human splanchnic amino-acid metabolism

**DOI:** 10.1007/s00726-016-2344-7

**Published:** 2016-10-06

**Authors:** Evelien P. J. G. Neis, S. Sabrkhany, I. Hundscheid, D. Schellekens, K. Lenaerts, S. W. Olde Damink, E. E. Blaak, C. H. C. Dejong, Sander S. Rensen

**Affiliations:** 1 Top Institute Food and Nutrition, P.O. Box 557, 6700 AN Wageningen, The Netherlands; 2 Department of General Surgery, NUTRIM School for Nutrition and Translational Research in Metabolism, Maastricht University, Universiteitssingel 50, 6229 ER Maastricht, The Netherlands; 3 CARIM School for Cardiovascular Diseases, Maastricht University Medical Centre, P.O. Box 5800, 6229 HX Maastricht, The Netherlands; 4 Department of Human Biology, NUTRIM School for Nutrition and Translational Research in Metabolism, Maastricht University, 6229 ER Maastricht, The Netherlands; 5 GROW School for Oncology and Developmental Biology, Maastricht University, P.O. Box 616, 6200 MD Maastricht, The Netherlands

**Keywords:** Amino acids, Interorgan metabolism, Gut microbiota, Obesity, Type 2 diabetes

## Abstract

Plasma levels of several amino acids are correlated with metabolic dysregulation in obesity and type 2 diabetes. To increase our understanding of human amino-acid metabolism, we aimed to determine splanchnic interorgan amino-acid handling. Twenty patients planned to undergo a pylorus preserving pancreatico-duodenectomy were included in this study. Blood was sampled from the portal vein, hepatic vein, superior mesenteric vein, inferior mesenteric vein, splenic vein, renal vein, and the radial artery during surgery. The difference between arterial and venous concentrations of 21 amino acids was determined using liquid chromatography as a measure of amino-acid metabolism across a given organ. Whereas glutamine was significantly taken up by the small intestine (121.0 ± 23.8 µmol/L; *P* < 0.0001), citrulline was released (−36.1 ± 4.6 µmol/L; *P* < 0.0001). This, however, was not seen for the colon. Interestingly, the liver showed a small, but a significant uptake of citrulline from the circulation (4.8 ± 1.6 µmol/L; *P* = 0.0138) next to many other amino acids. The kidneys showed a marked release of serine and alanine into the circulation (−58.0 ± 4.4 µmol/L and −61.8 ± 5.2 µmol/L, *P* < 0.0001), and a smaller, but statistically significant release of tyrosine (−12.0 ± 1.3 µmol/L, *P* < 0.0001). The spleen only released taurine (−9.6 ± 3.3 µmol/L; *P* = 0.0078). Simultaneous blood sampling in different veins provides unique qualitative and quantitative information on integrative amino-acid physiology, and reveals that the well-known intestinal glutamine–citrulline pathway appears to be functional in the small intestine but not in the colon.

## Introduction

Gut microbiota are increasingly implicated in the pathogenesis of obesity and type 2 diabetes. The functional output of the gut microbiota, in particular short-chain fatty acids and amino acids, seems to affect metabolic homeostasis profoundly (Neis et al. 2015; Wang et al. 2011). Gut bacteria partly determine the bioavailability of amino acids to the host by influencing dietary substrate metabolism and by directly providing amino acids to the host for uptake (Metges 2000). Indeed, the human distal gut microbiome contains a variety of gene clusters involved in amino-acid biosynthesis (Gill et al. 2006). Interestingly, several amino acids released by gut bacteria can serve as precursors for the synthesis of short-chain fatty acids (Barker 1981) which, in turn, appear to contribute to body weight gain (Schwiertz et al. 2010). In view of this, and considering the association between plasma levels of certain amino acids and metabolic dysregulation in obesity and type 2 diabetes (McCormack et al. 2013; Newgard 2012; Newgard et al. 2009), detailed knowledge on human splanchnic amino-acid metabolism is critical to understanding overall metabolic regulation.

Various body compartments have been demonstrated to play specialized roles in amino-acid homeostasis, taking up or releasing specific amino acids (Tizianello et al. 1980). However, the separate roles of the splanchnic organs in amino-acid metabolism in humans remain unclear. This is largely attributable to the inaccessibility of these organs and their efferent vessels (van de Poll et al. 2007b). As a result, most available human data concern overall splanchnic balances, which include contributions from all organs in the splanchnic bed. Only few human studies have been able to differentiate between the different intestinal segments (Fujita and Yanaga 2007; Olde Damink et al. 2002).

In this study, we aimed to provide a comprehensive overview of human splanchnic amino-acid metabolism, studying not only the liver and the kidneys, but also the spleen as well as the small versus the large intestine. We hypothesized that amino-acid metabolism of the small vs. the large intestine differs given the impact of gut bacteria on amino-acid bioavailability and their uneven distribution over the intestine. We show that the well-known intestinal glutamine–citrulline pathway appears to be present in the small intestine but not in the colon in vivo. This knowledge may be applied to optimize dietary supplementation (Iwasa et al. 2010; Petzke et al. 2014; Zhang et al. 2007) of certain amino acids and can be used for theoretical modelling of amino-acid metabolism in humans.

## Materials and methods

### Patients

Patients planned to undergo a pylorus preserving pancreatico-duodenectomy (PPPD) as a treatment for benign or malignant tumors were invited to participate in this study at the surgical outpatient clinic of Maastricht University Medical Center (MUMC^+^). All patients were pre-enrolled before surgery after giving written informed consent. Patients with known parenchymal and/or inflammatory liver disease as well as inflammatory bowel disease, inborn errors of metabolism, and/or use of antibiotics prior to surgery were excluded from the study. Moreover, excessive drinking (>20 alcoholic consumptions per week) and/or excessive smoking (>20 cigarettes per week) were exclusion criteria. All patients were on a stable Western diet before surgery except for two patients who received nutrient drinks to supplement their nutritional needs.

Permission for the study was granted by the local Medical Ethics Committee of Maastricht University Medical Center (MEC 11-3-084) and the study was conducted according to the ethical standards of the Helsinki Declaration of 1975 and in accordance with the Medical Research Involving Human Subjects Act (WMO).

### Study protocol

Anaesthesia was applied according to institutional routines as previously described (van de Poll et al. 2007b). During surgery, blood was sampled from the portal vein, one hepatic vein, the superior mesenteric vein, the inferior mesenteric vein, the splenic vein, and the right renal vein by direct puncture using 25G needles. Simultaneously, an arterial blood sample was drawn from the radial artery catheter.

### Amino-acid analysis

Upon sampling, blood was transferred to pre-chilled heparinized vacuum tubes (Becton-Dickinson Vacutainer, Franklin Lakes, NJ, USA) and centrifuged at 4 °C at 3500 rpm (r/mm = 200) for 10 min (Centrifuge 5417C, Eppendorf, Hamburg, Germany) to fractionate whole blood. The resulting plasma was transferred into Eppendorf cups, immediately frozen in liquid nitrogen, and stored at −80 °C. Before the amino-acid analysis, samples were thawed at 4 °C, whereupon the plasma samples were deproteinized with 5-sulfosalicylic acid (5-SSA) (4 mg/100 µL plasma), vortexed vigorously, and centrifuged at 4 °C at 23300 rpm in a Heraeus Biofuge Stratos centrifuge for 20 min. Ten microliters of the clear supernatant together with 10 µL of a 500-µM norvaline solution as internal standard were diluted with 980-µL ice-cold water in a 1.2-mL WISP vial and placed in the cooled sample-storage compartment of the WISP 712B sample processor (van Eijk et al. 1993).

### Equipment

The HPLC system consisted of two Jasco Model PU-980 pumps, a Model 717 plus autosampler from Gilson, and a Spark column oven from Waters (Etten-Leur, The Netherlands). The injection valve was equipped with a 20-µL sample loading loop. Analyses were performed on a 3-µm Microsphere C18 Bisschoff Spherisorb ODS II column (Felig et al. 1970) set at 21 °C (Waters, Etten-Leur, The Netherlands). The 5-µm Microsphere C18 Allsphere ODS II pre-column [7.5 mm × 4.6 mm (i.d.)] was filled with the same packing material. For the automated pre-column derivatization, a WISP 712B sample processor was used, equipped with a cooled sample-storage compartment which can be loaded with up to 48 samples in capped vials. Peak monitoring was performed with a Model FP 1520 fluorescence detector equipped with a xenon lamp and a 12-µL flow-cell (Jasco Benelux, Utrecht, The Netherlands). Detection was performed using an excitation wavelength of 330 nm and an emission cut-off filter of 440 nm (van Eijk et al. 1993). The coefficient of variance for all analyses was less than 4 %.

### Reagents and solvents

*o*-Phthalaldehyde (OPA) was obtained from Fisher Scientific (Breda, The Netherlands) and 3-mercaptopropionic acid (3-MPA) from Sigma-Aldrich (Zwijndrecht, The Netherlands). The OPA-MPA derivatization reagent was prepared by dissolving 15 mg of OPA in 0.5 mL of methanol, followed by adding 3.5 mL of potassium borate buffer (1.0 M, pH 10.08) and 15 µL of 3-MPA. ULC/MS grade methanol and acetonitrile were supplied by Biosolve (Valkenswaard, The Netherlands). An internal standard working solution was prepared by diluting 10-mM norvaline in 50-mM hydrochloric acid. We used HPLC-grade water processed with a Milli-Q UF Plus water purification system (Millipore, Waters). Individual amino acids were obtained from Sigma-Aldrich (Zwijndrecht, The Netherlands) and Merck (Schiphol-Rijk, The Netherlands). Amino-acid standard solutions were prepared by dissolving pure amino acids in ULC/MS grade water to a final concentration of 250 µM for each amino acid. Solvents used were of chromatographic grade. Solvent A consisted of 3.88-L 25-mM citric acid, adjusted to pH 6.8 with sodium hydroxide, containing 120 mL of tetrahydrofuran (THF) (Sigma-Aldrich, Zwijndrecht, The Netherlands). Solvent B was prepared by mixing 550-mL 25-mM citric acid, 400-mL acetonitrile, and 60-mL THF.

### Data processing

Amino-acid concentrations were calculated using the peak area relative to the area of the internal standard peak using the TotalChrom™ Chromatography Data System from Perkin Elmer instruments (Massachusetts, USA).

### Arterial venous differences

To quantify the contribution of the small intestine, colon, portal drained viscera (PDV), splanchnic area, liver, spleen, and kidneys in producing or extracting amino acids, we calculated arterial venous differences (ΔAV) as follows: ΔAV SMV = [*A*] − [SMV], ΔAV IMV = [*A*] − [IMV], ∆AV PDV = [*A*] − [PV], ∆AV splanchnic area = [*A*] − [HV], ∆AV liver = [*A* + 0.7 × PV] − 0.3 × [HV], ΔAV SV = [*A*] − [SV], and ΔAV RV = [*A*] − [RV] (Vrieze et al. 2012). In these equations, [SMV], [IMV], [PV], [HV], [SV], [RV], and [*A*] (Bouby et al. 1993) indicate superior mesenteric venous, inferior mesenteric venous, portal venous, hepatic venous, splenic venous, renal venous, and arterial concentrations, respectively. Positive arterial venous differences indicate net amino-acid uptake, whilst negative arterial venous differences indicate net release.

### Fractional extraction calculations

Fractional extraction of amino acids was calculated as [(*A*) − (*V*)]/*A* × 100, where *A* and *V* are the concentrations of amino acids in arterial and venous plasma, respectively. This calculation represents the percentage of amino-acid influx that is actually taken up from the bloodstream (Garibotto et al. 2003; van de Poll et al. 2007b).

### Statistics

Results are presented as means (SEM). The non-parametric Friedman test with Dunn’s Multiple Comparison Tests matched per subject was used for assessing statistically significant differences between arterial and venous concentrations of each amino acid of interest using GraphPad Prism version 5.01 for Windows (GraphPad Prism Software, Inc., USA). The strength of the linear association between AV differences of different amino acids was determined using the Spearman correlation coefficient. *P* values below 0.05 were considered statistically significant.

## Results

Twenty patients were enrolled in this study. Patient characteristics are shown in Table 1. Patients had a mean age of 64 years and a mean body mass index (BMI) of 24.7 kg/m^2^; 40 % was female.

**Table 1:** Baseline characteristics (*n* = 20)

Sex	
Male	12
Female	8
Age (years)	64 (54–75)
BMI	24.7 (18.7–29.3)
Plasma flows (mL/min)	
Portal vein	320 (42)
Hepatic artery	110 (23)
Splanchnic	430 (47)
Renal	606 (112)

*BMI* body mass index

### Absolute amino-acid concentrations in different abdominal veins

Table 2 shows the absolute arterial and venous plasma concentrations of 21 amino acids. The arterial concentrations we measured in this study were comparable to those we previously published for a group of pre-operative elective surgical patients (Dejong et al. 1996). In each blood vessel, glutamine was the most concentrated amino acid, with means ranging from 393.3 ± 5.3 µmol/L in the superior mesenteric vein to 518.1 ± 20.0 µmol/L in the radial artery. α-Aminobutyric acid, on the other hand, showed the lowest concentration, with the splenic vein displaying a lowest mean concentration of 18.1 ± 1.1 µmol/L. Table 3 shows the arterial venous differences of the 21 amino acids measured for the small intestine, colon, portal drained viscera (PDV), splanchnic area, liver, spleen, and kidneys.

**Table 2:** Absolute arterial and venous amino-acid concentrations (n = 20)

Concentrations (µmol/L)	Radial artery	Hepatic vein	Portal vein	Superior mesenteric vein	Inferior mesenteric vein	Splenic vein	Renal vein
Glutamate	56.4 (6.5)	122.4 (15.8)	54.5 (6.8)	61.1 (10.0)	49.1 (13.9)	43.6 (5.6)	61.3 (7.6)
Asparagine	46.0 (3.0)	41.6 (3.1)	50.5 (3.8)	50.7 (6.7)	46.4 (6.7)	49.3 (3.7)	44.1 (2.1)
Serine	97.2 (4.7)	92.0 (5.3)	98.6 (3.6)	101.8 (6.0)	93.4 (5.1)	94.1 (4.6)	130.0 (8.4)
Glutamine	518.1 (20.0)	485.0 (20.3)	481.2 (18.7)	393.3 (5.3)	497.7 (4.2)	500.0 (18.8)	509.8 (21.3)
Histidine	68.8 (2.6)	65.0 (2.8)	73.1 (2.7)	72.7 (4.9)	71.3 (5.0)	72.3 (3.3)	71.9 (2.7)
Glycine	236.9 (20.3)	258.0 (33.3)	256.9 (27.9)	291.9 (12.2)	236.6 (12.3)	249.5 (32.0)	247.7 (35.2)
Threonine	112.6 (6.2)	107.2 (6.0)	118.4 (6.5)	114.7 (4.8)	110.8 (6.8)	109.0 (5.1)	119.9 (8.2)
Citrulline	28.8 (2.8)	34.6 (3.4)	44.2 (4.0)	65.2 (8.9)	30.4 (13.7)	30.0 (2.8)	22.3 (1.9)
Arginine	65.3 (5.1)	49.3 (5.8)	75.5 (4.7)	83.6 (5.9)	68.2 (4.9)	66.2 (4.6)	73.9 (27.3)
Alanine	254.7 (22.0)	189.9 (18.5)	307.1 (23.7)	323.2 (26)	284.0 (23.0)	265.0 (22.0)	294.8 (8.2)
Taurine	63.4 (5.3)	67.0 (3.8)	70.5 (6.4)	67.4 (6.4)	71.1 (9.0)	73.6 (8.0)	61.2 (5.2)
aaba	18.4 (1.1)	18.5 (1.1)	18.3 (1.1)	18.8 (6.1)	18.5 (5.8)	18.1 (1.1)	18.9 (1.3)
Tyrosine	52.1 (3.3)	44.4 (3.0)	50.6 (3.1)	54.9 (6.8)	52.5 (6.1)	51.7 (4.1)	58.0 (4.0)
Valine	188.8 (7.3)	190.0 (7.5)	194.7 (6.8)	201.0 (4.0)	187.4 (3.9)	193.2 (8.3)	185.2 (8.3)
Methionine	24.2 (1.6)	19.1 (1.6)	25.6 (1.1)	27.8 (8.4)	23.6 (6.4)	23.5 (1.5)	22.4 (1.9)
Isoleucine	63.9 (4.2)	63.6 (4.5)	66.7 (4.0)	68.8 (5.4)	65.2 (6.1)	61.5 (4.0)	62.3 (4.8)
Phenylalanine	52.4 (2.0)	49.7 (2.1)	56.1 (1.7)	56.4 (4.3)	55.0 (4.1)	54.9 (2.1)	53.2 (2.2)
Tryptophan	30.1 (1.8)	28.9 (1.7)	29.4 (1.6)	30.5 (5.1)	28.8 (5.5)	27.3 (1.3)	29.2 (1.6)
Leucine	114.3 (6.0)	115.0 (6.5)	124.3 (5.4)	124.4 (5.4)	116.6 (5.5)	122.8 (6.8)	118.4 (6.8)
Ornithine	63.1 (5.7)	76.3 (7.4)	61.5 (4.9)	58.3 (8.8)	61.4 (8.6)	67.3 (5.9)	73.1 (8.3)
Lysine	137.9 (7.8)	135.5 (6.4)	159.1 (6.8)	159.9 (4.7)	152.0 (5.0)	149.3 (5.5)	154.3 (6.9)

Results are presented as means (SEM)

*aaba* α-aminobutyric acid

**Table 3:** Amino-acid handling by abdominal organs (*n* = 20)

Arterial venous differences (µmol/L)	Splanchnic area	PDV	Liver	Small intestine	Colon	Spleen	Kidneys
Glutamate	−66.4 (13.1)*	2.4 (3.6)	−74.0 (13.1)*	−5.0 (4.2)	6.8 (2.8)	12.4 (3.2)	−1.5 (3.2)
Asparagine	4.7 (1.3)	−4.4 (1.3)*	8.4 (1.8)*	−4.0 (1.5)*	−0.2 (1.1)	−3.0 (2.2)	−5.5 (0.8)
Serine	4.6 (2.8)	0.6 (1.7)	4.6 (2.8)	−5.0 (5.3)	4.3 (1.8)	2.6 (1.8)	−58.0 (4.4)*
Glutamine	29.3 (7.3)	42.1 (8.7)*	1.3 (6.2)	121.0 (23.8)*	20.8 (13.3)	14.3 (10.5)	22.2 (17.5)
Histidine	3.1 (1.5)	−3.2 (1.2)	6.0 (1.5)*	−5.0 (2.2)	−2.6 (2.6)	−4.2 (2.4)	−5.9 (1.8)
Glycine	−24.0 (15.8)	−14.8 (20.9)*	−14.2 (13.4)	−57.9 (22.4)*	−10.8 (17.2)	−15.5 (20.6)	−0.8 (20.2)
Threonine	0.6 (3.0)	−3.4 (1.8)	3.2 (3.2)	−7.0 (3.3)	2.0 (2.7)	−1.1 (3.1)	−5.7 (3.2)
Citrulline	−5.4 (1.1)	−14.5 (2.1)*	4.8 (1.6)*	−36.1 (4.6)*	−2.4 (3.1)	−0.8 (1.1)	17.3 (2.0)
Arginine	16.5 (3.9)	−8.1 (2.1)*	21.8 (4.7)*	−17.7 (4.5)*	−0.8 (2.2)	−0.3 (2.3)	−14.6 (2.4)
Alanine	54.2 (11.9)	−47.1 (5.3)*	86.3 (12.5)*	−79.0 (10.1)*	−36.7 (4.6)*	−20.8 (6.1)	−61.8 (5.2)*
Taurine	−3.0 (3.5)	−6.3 (2.5)	1.8 (3.7)	−3.4 (2.7)	−7.4 (2.7)	−9.6 (3.3)*	8.3 (4.9)
aab	0.1 (0.6)	0.4 (0.5)	−0.2 (0.6)	−0.2 (0.4)	0.2 (0.5)	0.5 (0.6)	−1.7 (0.4)
Tyrosine	7.8 (1.8)*	2.2 (2.2)	6.8 (1.7)*	−2.7 (2.3)	0.0 (1.5)	0.5 (2.8)	−12.0 (1.3)*
Valine	1.3 (3.8)	−1.8 (2.9)	3.1 (3.3)	−9.7 (4.5)	1.8 (3.1)	−2.0 (5.1)	4.3 (5.2)
Methionine	4.9 (1.2)*	−0.8 (1.0)	5.9 (1.1)*	−3.7 (1.8)	0.9 (1.4)	0.5 (1.5)	5.4 (1.9)
Isoleucine	1.8 (1.5)	−0.8 (1.5)	2.2 (1.5)	−3.3 (1.8)	−0.6 (1.7)	3.9 (2.2)	1.5 (3.2)
Phenylalanine	2.7 (1.0)	−2.8 (1.1)	5.0 (1.0)*	−4.0 (1.8)	−2.2 (1.1)	−2.5 (1.9)	0.5 (1.9)
Tryptophan	1.4 (0.7)	1.0 (0.8)	0.7 (0.5)	−0.3 (0.9)	1.6 (1.5)	2.9 (1.2)	3.2 (1.6)
Leucine	1.2 (2.3)	−6.6 (1.8)*	6.2 (2.4)*	−8.2 (3.6)	−2.1 (2.0)	−6.7 (2.9)	−13.1 (2.1)
Ornithine	−12.4 (3.5)	3.0 (1.3)	−14.9 (3.8)*	5.6 (3.4)	1.2 (2.6)	−3.4 (3.5)	−14.1 (5.7)
Lysine	3.5 (8.6)	−20.1 (7.7)*	18.3 (5.1)*	−20.9 (9.1)	−14.1 (9.3)	−10.3 (8.7)	−30.3 (10.0)

Results are presented as means (SEM)

Positive arterial venous differences indicate net amino-acid uptake, negative arterial venous differences indicate net release

* *P* values below 0.05 were considered statistically significant

### Amino-acid handling by the small intestine

Arterial venous differences of the different amino acids through the small intestine are displayed in Fig. 1a. As expected, glutamine was taken up in large amounts by the small intestine with a mean of 121.0 ± 23.8 µmol/L, while citrulline was released (−36.1 ± 4.6 µmol/L, both *P* < 0.0001). Glycine, asparagine, arginine, and alanine were significantly released by the small intestine as well, with a mean release of −57.9 ± 22.4 µmol/L (*P* = 0.0006), −4.0 ± 1.5 µmol/L (*P* < 0.0001), −17.7 ± 4.5 µmol/L (*P* < 0.0001), and −79.0 ± 10.1 µmol/L (*P* < 0.0001), respectively.

**Fig. 1a:**
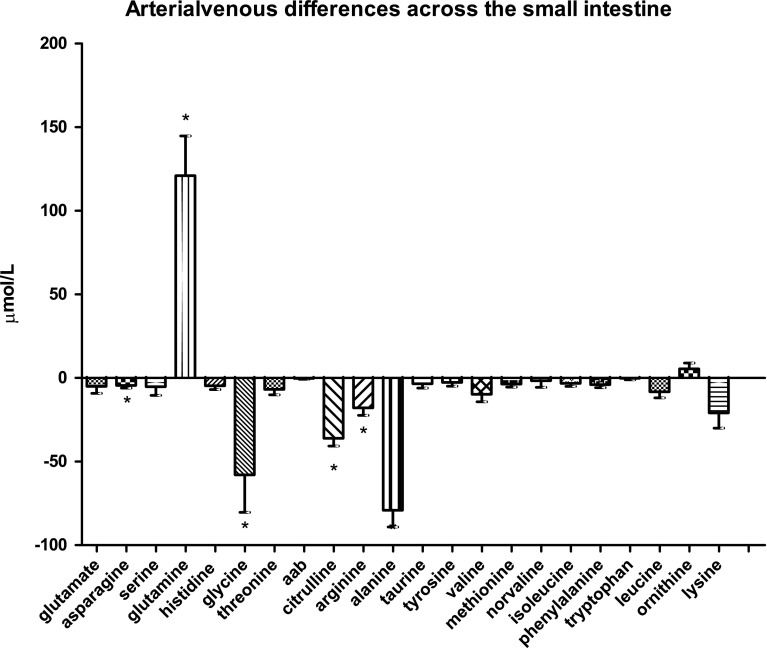
Arterial venous amino-acid concentration differences across the small intestine

### Amino-acid handling by the colon

We next analyzed the arterial venous differences of the different amino acids through the colon (Fig. 1b). In contrast to the small intestine, both glutamine uptake and citrulline release by the colon were not significant. The only significant difference that was found concerned alanine, which was released by the colon into the circulation with a mean of −36.7 ± 4.6 µmol/L (*P* < 0.0001). All other amino acids displayed only minor arterial venous differences.

### Amino-acid handling by the spleen

Among the 21 amino acids analyzed, only taurine showed a significant arterial venous difference across the spleen (−9.6 ± 3.3 µmol/L; *P* < 0.0145, Fig. 1c). Compared with the other organs, amino-acid handling in the spleen appeared to be much more variable among the different subjects tested.

**Fig. 1b:**
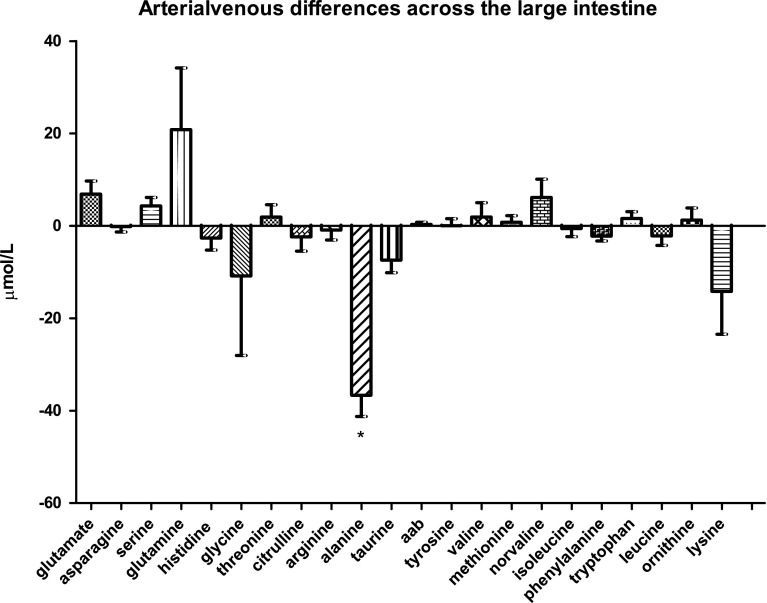
Arterial venous amino-acid concentration differences across the large intestine

### Amino-acid handling by the liver

The liver showed a small but significant uptake of citrulline from the circulation: 4.8 ± 1.6 µmol/L; *P* < 0.0138 (Fig. 1d). The percentage of citrulline influx that was actually taken up from the bloodstream equaled 12.1 %. Conversely, ornithine and especially glutamate were released by the liver into the circulation in larger quantities (−14.9 ± 3.8 µmol/L; *P* = 0.0011 and −74.0 ± 13.1 µmol/L; *P* = 0.0002, respectively). Many other amino acids were taken up by the liver to a small extent: asparagine (*P* = 0.0009), histidine (*P* = 0.0041), tyrosine (*P* = 0.0033), methionine (*P* = 0.0005), phenylalanine (*P* = 0.0011), and leucine (*P* = 0.0161). In contrast, alanine uptake by the liver was much more prominent, with an arterial venous difference of 86.3 ± 12.5 µmol/L, *P* = 0.0003.

### Amino-acid handling by the kidneys

The kidneys showed a marked release of serine and alanine into the circulation (−58.0 ± 4.4 µmol/L and −61.8 ± 5.2 µmol/L, *P* < 0.0001; Fig. 1e), and a smaller but statistically significant release of tyrosine (−12.0 ± 1.3 µmol/L, *P* < 0.0001). No amino acids were taken up by the kidneys to a significant extent.

**Fig. 1c:**
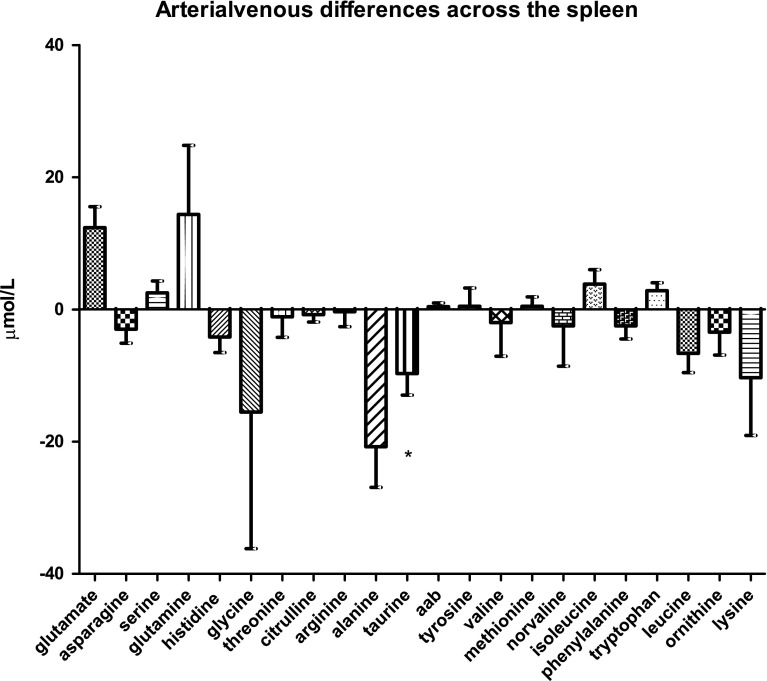
Arterial venous amino-acid concentration differences across the spleen

### Amino-acid handling by the portal drained viscera

Whereas glutamine was significantly taken up by the portal drained viscera with a mean of 42.1 ± 8.7 µmol/L (*P* < 0.0001), arginine, citrulline, asparagine, glycine, alanine (all *P* < 0.0001), leucine (*P* = 0.0014), and lysine were all significantly released by the portal drained viscera (Fig. 1f). The corresponding mean arterial venous differences were: −8.1 ± 2.1 µmol/L for arginine; −14.5 ± 2.1 µmol/L for citrulline; −4.4 ± 1.3 µmol/L for asparagine; −14.8 ± 20.9 µmol/L for glycine; −47.1 ± 5.3 µmol/L for alanine, −6.6 ± 1.8 µmol/L for leucine, and −20.1 ± 7.7 µmol/L for lysine.

**Fig. 1d:**
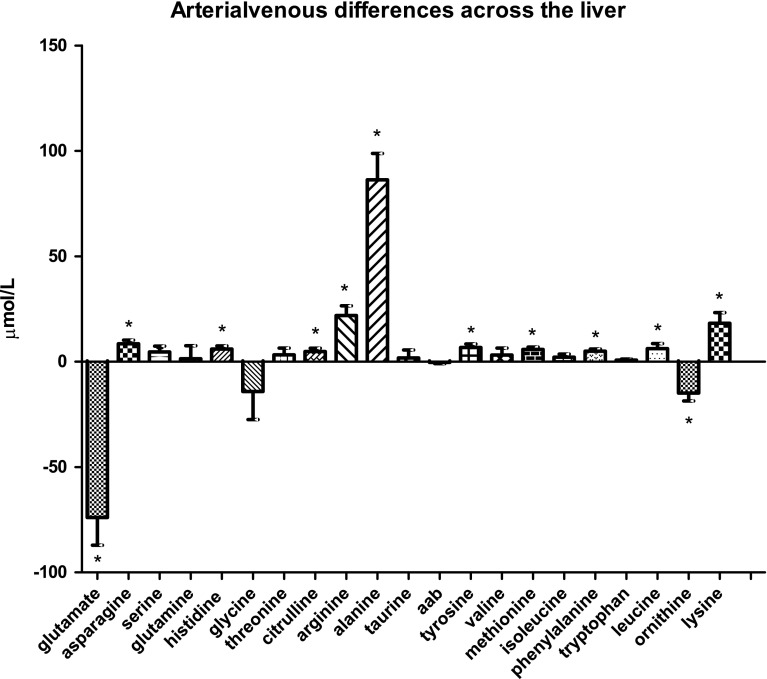
Arterial venous amino-acid concentration differences across the liver

### Amino-acid handling by the overall splanchnic area

The splanchnic area, including contributions from the liver as well as extrahepatic tissues (i.e., the gastrointestinal tract, spleen, and pancreas), released glutamate into the circulation (−66.4 ± 13.1 µmol/L; *P* < 0.0001, Fig. 1g). Conversely, tyrosine and methionine were the only amino acids to be taken up by the splanchnic area, albeit to a much lower extent; 7.8 ± 1.8 µmol/L and 4.9 ± 1.2 µmol/L, respectively (both *P* < 0.0001).

## Discussion

In this study, we have explored amino-acid handling by separate splanchnic organs. In view of the increasingly acknowledged role of the gut microbiota in amino-acid metabolism and energy metabolism, we payed special attention to the differences in amino-acid handling between the large intestine, which is densely populated with bacteria, and the small intestine, which contains less bacteria.

**Fig. 1e:**
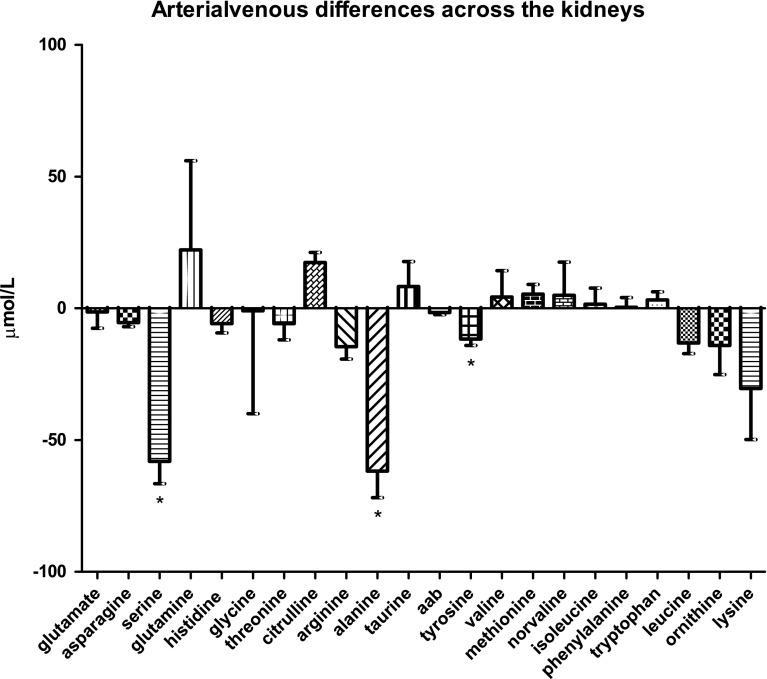
Arterial venous amino-acid concentration differences across the kidneys

 In line with pioneering observations in the isolated, vascularly perfused rat intestine (Windmueller et al. 1970) and from the intestine in vivo in a number of different animals (Windmueller 1982), we found that glutamine was taken up in large amounts by the human small intestine. Interestingly, in these rat models, it seemed that the intestinal utilization of glutamine was unrelated to the activities of the gut microbiota as glutamine extraction was quantitatively similar in germ-free rats compared with the conventional rats. However, the altered gut microbiota composition in obese animals (Backhed et al. 2004; Turnbaugh et al. 2006) may still influence intestinal glutamine metabolism, which has been shown to be altered in obesity (Osto et al. 2013; Wolff et al. 2009).

An important aspect of glutamine metabolism in the intestine is the resulting production of citrulline, which is the precursor of arginine synthesis in extrahepatic organs, including the kidneys (Wu et al. 1994). Consistent with observations in animal studies and in vivo studies in man (Fujita and Yanaga 2007; van de Poll et al. 2007a), we were able to find a positive association between glutamine extraction and citrulline release by the human small intestine. The endogenous conversion of glutamine into citrulline by the small intestine seems to be of clinical importance (Garlick 2001) as decreased glutamine concentrations in the intestinal mucosa are associated with mucosal atrophy, as well as an impaired intestinal barrier function and bacterial translocation (Ding and Li 2003; Rao and Samak 2012). All these features have been implicated in the pathogenesis of obesity-related conditions, such as type 2 diabetes and non-alcoholic steatohepatitis (Shaw et al. 2012). Hence, glutamine supplementation may be effective in these metabolic disorders as it has been in other conditions (Nose et al. 2010; Zhang and Jiang 2013).

We found that glutamine extraction occurred in the small intestine as well as the colon. However, the mean fractional extraction rate of glutamine was approximately six times higher in the small intestine (24.1 %) as compared with the colon (3.9 %). In accordance with earlier studies (Fujita and Yanaga 2007; Olde Damink et al. 2002), superior mesenteric venous citrulline levels were significantly higher than inferior mesenteric venous citrulline levels, implying that the small intestine is more important for the production of citrulline than the colon. Furthermore, the only amino acid to be released by the colon was alanine. Whereas the quantitative main production site of alanine in the body is muscle, colonic alanine release may contribute to endogenous glucose production in the liver as well. The finding that the colon did not show any other arterial venous differences is interesting in view of the suggested role for colonic bacteria in amino-acid metabolism (Neis et al. 2015). It is thought that amino acids are not significantly absorbed by the colonic mucosa, but rather intensively metabolized by the colonic microbiota (Davila et al. 2013). Partly, due to differences in microbiota abundance and composition along the gastrointestinal tract, bacterial amino-acid metabolism in the gut is likely to be compartment specific. In addition, nutritional status appears to play a role as well. Bacterial amino-acid metabolism might still benefit the host when food resources are scarce to enhance ecosystem stability (Costello et al. 2010).

Regarding the proposed interorgan relation between citrulline and arginine metabolism in the gut, the liver, and the kidneys (Houdijk et al. 1994), we were not able to demonstrate significant renal citrulline uptake or renal arginine release. Normally, arginine is released into the renal vein after being synthesized from citrulline taken up from the bloodstream (Chen and Baylis 2010; Dhanakoti et al. 1990). The fact that we only observed a slight non-significant renal citrulline uptake could be related to our relatively small data set. Interestingly, renal citrulline uptake was considerably higher than the rate at which citrulline was released from the splanchnic area, supporting that also other processes than glutamine metabolism provide citrulline for renal arginine synthesis (Castillo et al. 1996). In line with earlier findings in humans (Brosnan 1987; Tizianello et al. 1980, 1985), our data show a net renal release of serine into the systemic circulation. In contrast to our data, those studies also showed a significant renal uptake of glycine, which was interpreted as evidence for conversion of glycine to serine in the kidneys. A possible explanation for this discrepancy is that glycine is probably also supplied to the kidneys from sources other than direct uptake from the bloodstream (Brosnan 1987; Dejong et al. 1998; Pitts and MacLeod 1972) This, in turn, offers an additional explanation why glycine uptake accounts for only 30 % of the net renal release of serine (van de Poll et al. 2004). On average, 1.5 g glycine is taken up by the human kidneys per day (Tizianello et al. 1980), followed by a release of 4 g serine per day (Tizianello et al. 1985). Next to serine, the kidneys also released significant amounts of alanine and tyrosine in our test subjects. Prior studies already elucidated the conversion of phenylalanine to tyrosine and found that the kidney is the major source of circulating tyrosine as the kidneys alone would be capable of producing all the tyrosine needed by the body (van de Poll et al. 2004).

The general view of the liver being an organ which does not take up any citrulline was disproved by our data set. The fractional hepatic extraction of citrulline was 12.1 %. Although hepatic citrulline metabolism is still an unknown area, we can speculate that hepatic citrulline may serve for de novo arginine synthesis followed by nitric oxide synthesis. Glutamate, on the other hand, was significantly released by the liver into the circulation together with ornithine. It is well known that the liver contains high levels of arginase I, which converts approximately 15 % of plasma arginine to ornithine and urea, and, finally, to glutamate (Silva et al. 2006; Wu and Morris 1998). Enteral supplementation of arginine to support gut function has, therefore, already proven to be ineffective (Cynober 1994). Besides, hepatic arginine uptake also serves other purposes in the liver, including protein synthesis. As a result, the liver releases significant amounts of ornithine and urea, as confirmed in this study, whereas it does not release significant amounts of arginine. Since our study showed hepatic uptake of arginine accompanied by hepatic release of glutamate and to a smaller extent release of ornithine, we assume that arginine is partly degraded to glutamate in the liver, as supported by other studies (O’Sullivan et al. 1998; Wu and Morris 1998).

Finally, we found that the spleen released taurine into the circulation. Taurine, which is the most abundant amino acid in white blood cells, especially reaches high concentrations in tissues with a high oxidative activity and in tissues exposed to increased levels of oxidizing agents, including the spleen and kidneys (Marcinkiewicz and Kontny 2014). Since the spleen contains a high number of lymphocytes, the pronounced release of taurine by the spleen might reflect lymphocyte degradation, or taurine release by lymphocytes.

**Fig. 1f:**
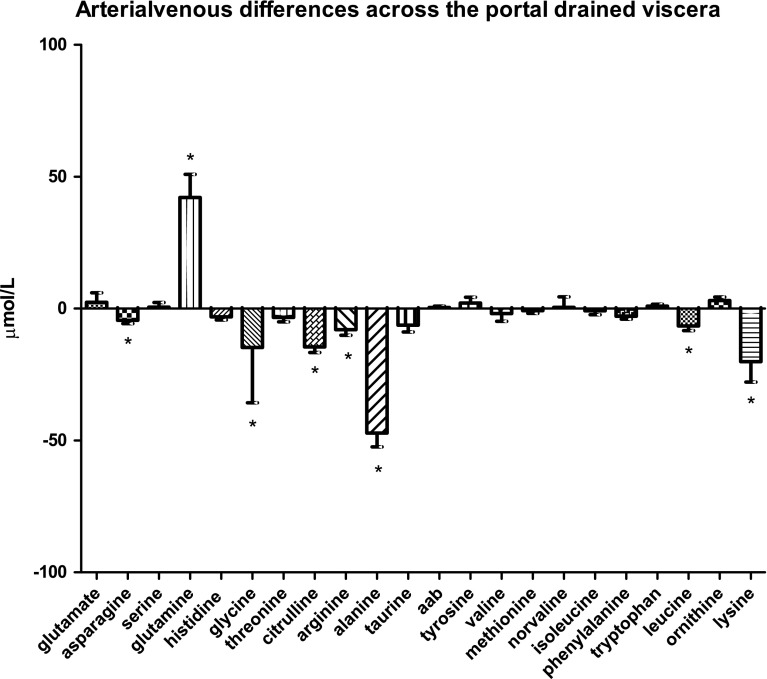
Arterial venous amino-acid concentration differences across the portal drained viscera

**Fig. 1g:**
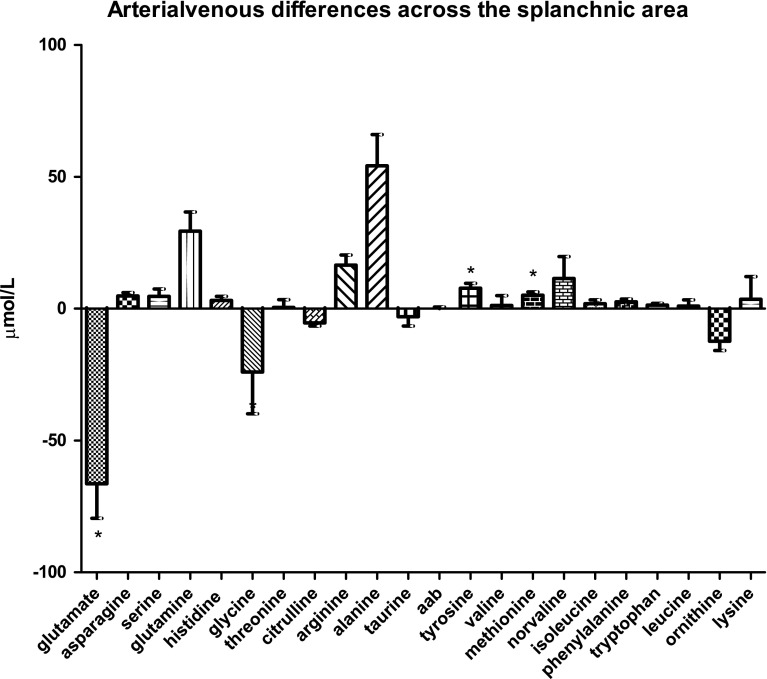
Arterial venous amino-acid concentration differences across the splanchnic area

## Conclusion

In conclusion, we show that the well-known intestinal glutamine–citrulline pathway appears to be present in the human small intestine but not in the colon in vivo. Another interesting observation from this study is the significant release of taurine by the spleen. In relation to nutrition research, these additions to our knowledge on the physiology of human amino-acid metabolism are important to develop (organ) specific nutritional support. Furthermore, in light of the health claims of pre- and probiotics, a detailed picture of the physiology of human amino-acid metabolism as provided in this study, its interaction with the microbiome, as well as the effects manipulation by altering the diet or the composition of the microbiota is of pivotal importance.

## Abbreviations

PPPD: Pylorus-preserving pancreatico-duodenectomy
BMI: Body mass index
5-SSA: 5-Sulfosalicylic acid
HPLC: High-performance liquid chromatography
OPA: *o*-Phthalaldehyde
3-MPA: 3-Mercaptopropionic acid
THF: Tetrahydrofuran
